# Ablação por Campo Pulsado versus Ablação por Radiofrequência de Curta Duração e Potência Muito Alta na Fibrilação Atrial: Uma Revisão Sistemática e Metanálise

**DOI:** 10.36660/abc.20240845

**Published:** 2025-07-29

**Authors:** Asad Iqbal, Wellgner Fernandes Oliveira Amador, Martin Cevallos-Cueva, Maaz Ahmad, Juan Alejandro Pinilla Alarcón, Radamés Vieira Diniz

**Affiliations:** 1 Bacha Khan Medical College Mardan Paquistão Bacha Khan Medical College, Mardan – Paquistão; 2 Universidade Federal de Campina Grande Cajazeiras PB Brasil Universidade Federal de Campina Grande, Cajazeiras, PB – Brasil; 3 School of Medicine Universidad Central del Ecuador Quito Equador School of Medicine, Universidad Central del Ecuador, Quito – Equador; 4 CES University, School of Medicine Medellín Colômbia CES University, School of Medicine, Medellín – Colômbia

**Keywords:** Ablação por Cateter, Fibrilação Atrial, Eficácia, Segurança

## Abstract

**Fundamento:**

O isolamento das veias pulmonares (IVP) é um tratamento fundamental para a fibrilação atrial (FA). A ablação por campo pulsado (PFA) e a ablação por radiofrequência (RF) de curta duração e potência muito alta (vHPSD) são tecnologias emergentes, mas sua eficácia e segurança comparativas ainda não estão claras.

**Objetivos:**

Avaliar a eficácia e a segurança da PFA em comparação à ablação por RF vHPSD para FA.

**Métodos:**

Uma busca sistemática nas bases de dados PubMed, Embase, Web of Science e Cochrane identificou estudos comparando a ablação por PFA e vHPSD. Os desfechos incluíram sucesso da IVP, tempo de procedimento pele a pele, tempo de fluoroscopia, ausência de arritmias atriais e complicações como tamponamento cardíaco, acidente vascular cerebral e eventos de acesso vascular. Os desfechos contínuos foram analisados por meio de diferenças médias (DM), enquanto os desfechos binários foram avaliados por razões de risco (RR) e intervalos de confiança (IC) de 95%. Um limiar de significância de p < 0,05 foi considerado para análises estatísticas. Este estudo está registrado no PROSPERO (CRD42024619301).

**Resultados:**

Quatro estudos observacionais com 605 pacientes foram incluídos, dos quais 315 (52%) foram submetidos a PFA. PFA e vHPSD alcançaram sucesso de IVP semelhante (RR 1,00; IC 95% 0,99–1,01; p = 1). PFA reduziu o tempo do procedimento (MD -30,07 min; IC 95% -31,41 a -28,74; p < 0,01), mas aumentou o tempo de fluoroscopia (MD 6,87 min; IC 95% 3,66–10,08; p < 0,01). A ausência de arritmias atriais foi comparável (RR 1,03; IC 95% 0,94–1,14; p = 0,5). As taxas de complicações, incluindo tamponamento cardíaco, acidente vascular cerebral e problemas de acesso vascular, não mostraram diferenças significativas entre os grupos.

**Conclusão:**

A PFA reduz significativamente o tempo do procedimento, mas requer fluoroscopia mais longa em comparação com a vHPSD. Ambas as técnicas apresentam eficácia comparável para IVP e ausência de arritmias, com perfis de segurança semelhantes.

## Graphical abstract

**Figura Central:**
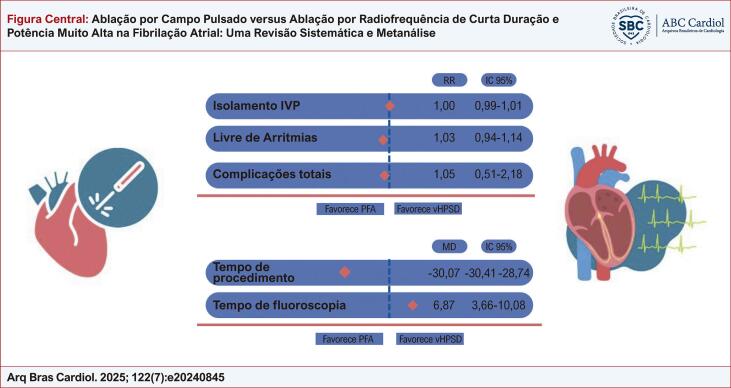
Ablação por Campo Pulsado versus Ablação por Radiofrequência de Curta Duração e Potência Muito Alta na Fibrilação Atrial: Uma Revisão Sistemática e Metanálise

## Introdução

A fibrilação atrial (FA), a arritmia cardíaca sustentada mais comum, impacta significativamente os cuidados de saúde globais devido à sua associação com o aumento da morbidade, mortalidade e utilização dos cuidados de saúde.^1,2^ A ablação por cateter surgiu como um tratamento fundamental para a FA, oferecendo controle do ritmo e alívio sintomático.^2,3^ Entre as estratégias de ablação contemporâneas, a ablação por radiofrequência (RF) de curta duração e potência muito alta (vHPSD) é valorizada por sua eficiência e precisão, enquanto a ablação por campo pulsado (PFA), uma técnica não térmica que utiliza eletroporação, ganhou atenção por seu potencial para minimizar danos colaterais aos tecidos.^4-6^

Desde a sua introdução na Europa em 2021, a PFA tem sido cada vez mais adotada devido ao seu promissor perfil de segurança, particularmente na redução do risco de complicações como estenose da veia pulmonar e danos às estruturas adjacentes.^7^ No entanto, apesar desses avanços, ainda há uma escassez de comparações diretas entre a ablação por RF de PFA e vHPSD, uma vez que os estudos existentes são em grande parte observacionais e retrospectivos por natureza.^8-11^ Essa falta de ensaios clínicos randomizados limita a capacidade de estabelecer perfis comparativos definitivos de eficácia e segurança.

Para abordar essa lacuna, esta metanálise sintetiza sistematicamente as evidências disponíveis para avaliar os desfechos clínicos e processuais associados à ablação por RF com PFA e vHPSD. Ao fornecer uma avaliação abrangente dessas técnicas, incluindo a análise sequencial de ensaios clínicos (TSA), este estudo busca subsidiar a tomada de decisões clínicas e apoiar a otimização de estratégias de ablação para o manejo da FA em diversas populações de pacientes. Escolhemos a ablação por RF vHPSD como comparador porque é a evolução mais recente da ablação térmica, diferindo da HPSD por utilizar aplicações ultracurtas e de alta energia com controle ativo de temperatura.

## Métodos

Esta revisão sistemática seguiu as recomendações da Colaboração Cochrane^12^ e as diretrizes de Itens de Relatório Preferenciais para Revisões Sistemáticas e Meta-Análises (PRISMA),^13^ incluindo o delineamento, a implementação das etapas, a análise e a descrição dos resultados. O protocolo do estudo foi registrado no Registro Prospectivo Internacional de Revisões Sistemáticas (PROSPERO) sob o número CRD42024619301.

### Estratégia de busca

Uma busca sistemática nas bases de dados PubMed (MEDLINE), Embase, Cochrane Central e Clinical Trial foi realizada em 25 de novembro de 2024. Os seguintes termos de cabeçalhos de assuntos médicos foram incluídos: ‘atrial fibrillation’, ‘af’, ‘afib’, ‘atrial fibrillation’, ‘a-fib’, ‘atrial flutter’, ‘cardiac arrhythmias’, ‘catheter ablation’, ‘ablation’, ‘pulsed field’, ‘pfa’, ‘hpsd’, ‘high power short duration’. A estratégia de busca é detalhada na Tabela Suplementar S1.

### Extração de dados

Após a remoção das duplicatas, dois autores (A.I. e W.A.) selecionaram os títulos e resumos, avaliando independentemente os artigos completos para inclusão com base em critérios pré-especificados. Discrepâncias foram discutidas e resolvidas por um terceiro revisor (M.A.). A extração de dados foi conduzida independentemente por A.I. e W.A., priorizando as informações relevantes para o objetivo do estudo.

### Critérios de elegibilidade

Estudos elegíveis para esta revisão sistemática atenderam aos seguintes critérios: (I) estudos avaliando eficácia ou segurança sem restrições de tempo; (II) inclusão de pacientes com FA; (III) intervenções envolvendo PFA; (IV) ablação de vHPSD como controle; e (V) relatar pelo menos um desfecho de interesse. Os critérios de exclusão foram: (I) populações sobrepostas, definidas por instituições compartilhadas e períodos de recrutamento; (II) populações fora do escopo de interesse; (III) literatura republicada; (IV) protocolos sem resultados relatados; (V) revisões, relatos de caso, séries de casos, artigos de referência, opiniões de especialistas ou estudos in vivo/in vitro; (VI) dados duplicados do mesmo ensaio clínico; ou (VII) ausência de um grupo comparador.

No presente estudo, a técnica de ablação por RF vHPSD foi definida com base em parâmetros estabelecidos na literatura.^4^ Consideramos vHPSD os procedimentos que utilizam potência ≥ 90W com duração ≤ 4 segundos por aplicação, realizados através de cateteres irrigados com controle de temperatura.

### Medidas de resultados e análise de subgrupos

Os resultados de eficácia foram: (1) sucesso na obtenção do isolamento da veia pulmonar, (2) tempo de procedimento pele a pele, (3) tempo de fluoroscopia, (4) ausência de qualquer arritmia atrial (recorrências de flutter atrial, FA e taquicardia atrial com duração de pelo menos 30 segundos durante o acompanhamento após um período de bloqueio de 1 mês), e (5) tempo de permanência no átrio esquerdo. Os desfechos de segurança incluíram (6) incidência geral de complicações, (7) tamponamento cardíaco, (8) reações no local de acesso vascular e (9) acidente vascular cerebral ou AIT.

### Avaliação de qualidade

Avaliamos o risco de viés usando o Cochrane ROBINS-I (Risco de viés em estudos não randomizados de intervenções) ferramenta,^14^ que avalia estudos não randomizados de intervenções em sete domínios: fatores de confusão, seleção de participantes, classificação das intervenções, desvios das intervenções pretendidas, dados ausentes, mensuração dos desfechos e seleção dos resultados relatados. A avaliação foi realizada de forma independente por dois revisores (A.I. e A.P.), com as discordâncias resolvidas por discussão ou consulta com um terceiro revisor. Cada domínio foi classificado como tendo risco de viés baixo, moderado, grave ou crítico, garantindo uma avaliação abrangente. O layout foi produzido por RobVis.^15^

### Certeza da evidência

Além disso, a ferramenta de Classificação de Recomendações, Avaliação, Desenvolvimento e Avaliação (GRADE) foi empregada por dois autores independentes (W.A. e M.A.) usando a ferramenta de desenvolvimento de diretrizes GRADEpro Guideline Development Tool^16^ paraavaliar o nível de certeza das evidências nesta metanálise, com categorizações que variam de alto a muito baixo.^17^ Quaisquer divergências foram discutidas e resolvidas por consenso.

### Análise de sensibilidade

A estabilidade das estimativas combinadas foi avaliada por meio de uma análise “leave-one-out”, na qual os dados de cada estudo foram removidos sequencialmente e o conjunto de dados restante foi reanalisado. Isso ajudou a garantir que nenhum estudo individual influenciasse indevidamente os tamanhos de efeito agregados.

### Análise estatística

A análise estatística foi realizada usando o software R e RStudio (versão 2024.04.1+748; R Core Team, Viena, Áustria), empregando o modelo de efeitos aleatórios de DerSimonian e Laird para calcular análises agrupadas com intervalos de confiança (IC) de 95%.^18^ Os resultados foram apresentados como uma análise conjunta em gráficos florestais. Os desfechos binários foram avaliados com razões de risco (RRs), os desfechos contínuos com diferenças médias (DMs), e os resultados foram apresentados em gráficos de floresta. A heterogeneidade foi avaliada pelo teste qui-quadrado de Cochrane Q e pela estatística I^2^, com valores de P < 0,10 e I^2^ > 30% indicando heterogeneidade significativa.^19^ O viés de publicação foi avaliado com gráficos de funil.

### Análise de metarregressão

Para avaliar a influência da proporção de pacientes com FA persistente nos desfechos clínicos e do procedimento, foram realizadas análises de metarregressão. A porcentagem de pacientes com FA persistente foi incluída como covariável no modelo para avaliar seu potencial impacto no tempo total de fluoroscopia e na ausência de arritmias atriais, que foram utilizadas como variáveis dependentes. As análises foram realizadas utilizando o software R (versão 4.4), com os resultados relatados como estimativas e intervalos de confiança de 95%. A significância estatística foi determinada com um limiar de p < 0,05.

### Análise sequencial de testes

A análise sequencial do ensaio (TSA) foi realizada usando o software TSA (versão 0.9.5.10 beta)^20^ para avaliar a adequação do tamanho da amostra e determinar a necessidade de pesquisas adicionais. O tamanho da informação ajustado pela diversidade foi calculado, considerando a variabilidade entre os ensaios e o erro amostral, com um risco de erro tipo I de 5% (α = 5%) e um risco de erro tipo II de 20% (Β = 20%).^21,22^ Cruzar o limite de monitoramento sequencial do teste antes de atingir o tamanho de informação necessário indica evidência conclusiva, enquanto não ultrapassá-lo sugere a necessidade de testes adicionais.

## Resultados

### Seleção de estudos

**Figura 1:**
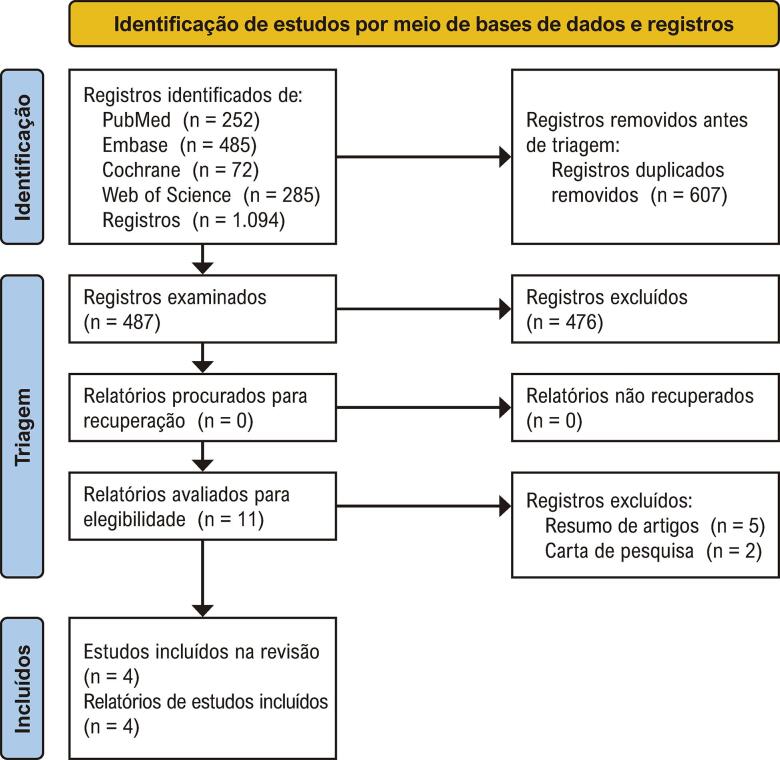
– Diagrama de fluxo de triagem e seleção de estudos de itens de relatórios preferenciais para revisões sistemáticas e metanálises (PRISMA).

A estratégia de busca inicial gerou 1.094 resultados (Figura 1). Após a remoção de 607 duplicatas, 487 artigos foram selecionados com base no título e no resumo, de acordo com os critérios de inclusão e exclusão estabelecidos. Desse conjunto, 11 registros foram selecionados para leitura completa. Por fim, esta metanálise incluiu quatro estudos observacionais retrospectivos.^8-11^

### Características basais dos estudos incluídos

Os estudos incluíram um total de 605 pacientes, dos quais 315 (52%) foram submetidos à PFA. O período de acompanhamento se estendeu por até 6 meses. A idade média variou de 61,6 a 67 anos, com 250 (41%) sendo do sexo feminino. Além disso, 234 (37%) dos participantes apresentavam FA persistente. O IMC variou de 25,23 a 28 kg/m^2^. As comorbidades mais comuns foram hipertensão (380, 62,8%), doença arterial coronariana (93, 17,3%) e diabetes mellitus (33, 12,5%). As características basais dos estudos incluídos estão detalhadas na Tabela 1.

**Tabela 1:** – Características basais dos estudos incluídos

Estudos	Acompanhamento (meses)	Tamanho da amostra		Feminino, n (%)		Idade, anos (DP)		FA persistente, n (%)		FA paroxística, n (%)		IMC, kg/m^2^ (DP)		FEVE, % (DP)		DM, n (%)		ESCORE CHA2DS2-VASc (DP)		IC congestiva, n (%)		DAC, n (%)		HTA, n (%)	
		PFA	vHPSD	PFA	vHPSD	PFA	vHPSD	PFA	vHPSD	PFA	vHPSD	PFA	vHPSD	PFA	vHPSD	PFA	vHPSD	PFA	vHPSD	PFA	vHPSD	PFA	vHPSD	PFA	vHPSD
Dello Russo 2024	6	171	171	64 (37)	60 (35)	64,3 (7,5)	65,3 (9)	56 (33)	54 (32)	115 (67)	117 (68)	25,23 (1,12)	25,23 (1,12)	57,4 (5,2)	58,2 (3,7)	N / D	N / D	2 (1,5)	2 (1,5)	46 (27)	41 (24)	24 (14)	22 (13)	105 (61)	106 (62)
South 2024	6	52	30	15 (29)	16 (53)	67 (10)	73 (7)	27 (52)	17 (57)	25 (48)	13 (43)	28 (6)	27 (6)	52 (8)	47 (14)	8 (15)	4 (13)	4 (1,6)	4 (1,7)	20 (38)	13 (43)	9 (17)	13 (43)	43 (83)	25 (83)
Wormann 2023	6	57	57	38 (67)	34 (60)	67 (13)	67 (12)	40 (70)	40 (70)	17 (30)	17 (30)	28 (5)	27 (4)	56 (6)	56 (9)	9 (16)	8 (14)	3 (N/D)	3 (N/D)	N/D	N/D	14 (25)	11 (19)	37 (65)	34 (60)
Popa 2023	6	35	32	11 (31)	12 (37,5)	61,6 (11,4)	62,2 (12,8)	0 (0)	0 (0)	35 (100)	32 (100)	26,7 (4,9)	25,4 (2,7)	58,5 (2,7)	57,9 (7,8)	1 (2,9)	3 (9,4)	1,7 (1,7)	2.0 (1.4)	N/D	N/D	N/D	N/D	14 (40)	16 (50)

Variáveis contínuas são apresentadas como média basal (Desvio Padrão, DP). N/D: não disponível; DP: desvio padrão; FA: fibrilação atrial; IMC: índice de massa corporal; FEVE: fração de ejeção do ventrículo esquerdo; DM: diabetes mellitus; IC: insuficiência cardíaca; DAC: doença arterial coronariana; HAS: hipertensão. Todos os estudos incluídos adotaram um nível de significância estatística de α = 0,05. Fonte: Autores.

### Análise conjunta de todos os estudos

**Figura 2:**
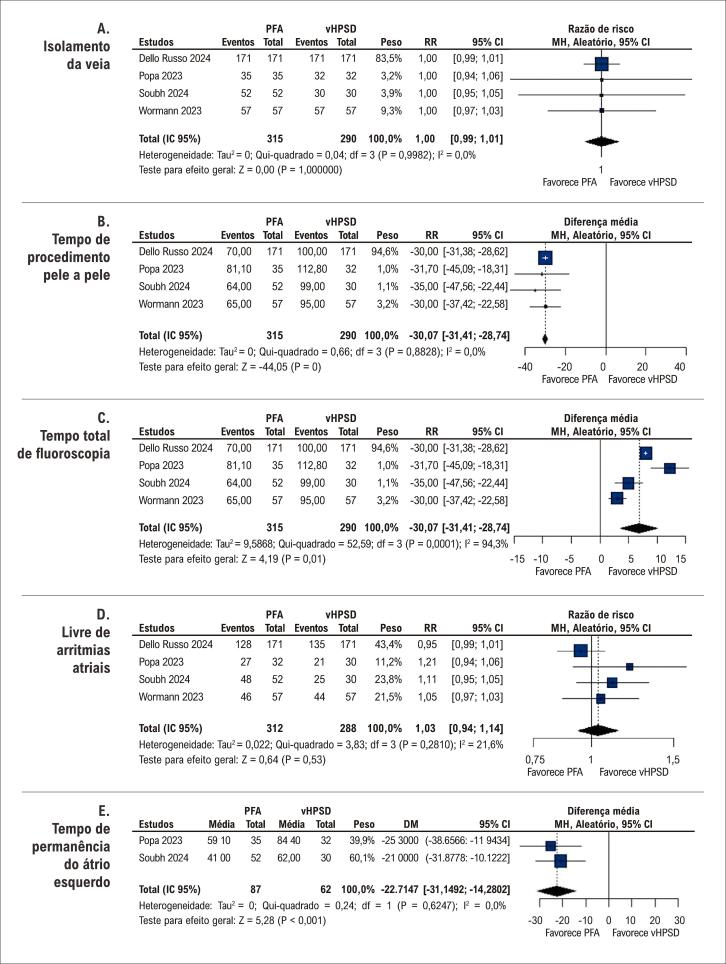
– Gráficos de floresta para (A) isolamento da veia pulmonar; (B) tempo de procedimento pele a pele; (C) tempo total de fluoroscopia; (D) ausência de arritmias atriais; e (E) tempo de permanência no átrio esquerdo. Fonte: Autores.

Uma análise conjunta de quatro estudos demonstrou que ambas as intervenções alcançaram o isolamento da veia pulmonar com eficácia igual (RR 1,00; IC 95% 0,99–1,01 [Figura 2A]). No entanto, o tempo do procedimento pele a pele foi maior no grupo de ablação com vHPSD (DM -31,41 min; IC 95% -31,41 a -28,74 [Figura 2B]), enquanto o tempo total de fluoroscopia foi maior no grupo PFA (DM 6,87 min; IC 95% 3,66–10,08 [Figura 2C]). Nenhuma diferença significativa foi observada na ausência de quaisquer arritmias atriais (RR 1,03; IC 95% 0,94–1,14 [Figura 2D]). Notavelmente, o tempo de permanência no átrio esquerdo favoreceu a intervenção com PFA (DM -22,15 min; IC 95% -31,15 a -14,3 [Figura 2E]).

**Figura 3:**
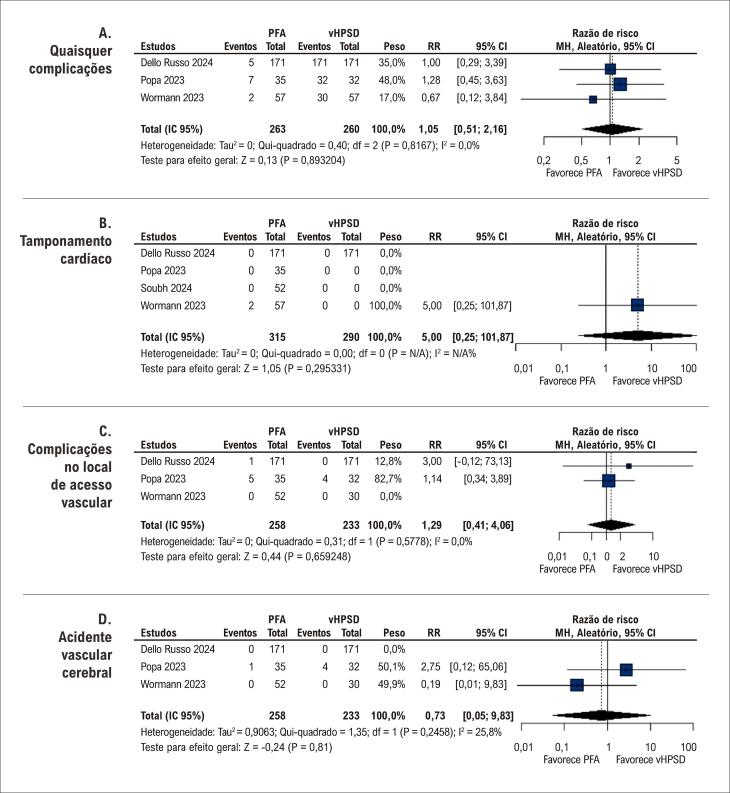
– Gráficos de floresta para (A) quaisquer complicações; (B) tamponamento cardíaco; (C) complicações no local de acesso***.***

Os perfis de segurança das duas intervenções foram comparáveis. Não houve diferenças significativas na incidência de complicações gerais (RR 1,05; IC 95% 0,51-2,16 [Figura 3A]), tamponamento cardíaco (RR 5,00; IC 95% 0,25-101,87 [Figura 3B]), reações no local de acesso vascular (RR 1,29; IC 95% 0,41-4,06 [Figura 3C]) ou acidente vascular cerebral (RR 0,73; IC 95% 0,05-9,83 [Figura 3D]).

### Risco de viés nos estudos

Conforme detalhado na Figura Suplementar S1, três estudos^8-10^ foram avaliados como tendo um risco moderado de viés. Isso foi atribuído predominantemente a problemas de confusão, seleção de participantes, classificação da intervenção e mensuração de resultados. Um estudo^11^ foi categorizado como tendo alto risco de viés, principalmente devido à confusão.

### Certeza da evidência e viés de publicação

De acordo com os critérios GRADE (Tabela Suplementar S2), a certeza da evidência foi muito baixa para todos os desfechos avaliados. Isso foi atribuído principalmente ao desenho não randomizado dos estudos incluídos e ao seu risco de viés moderado a alto. A análise do gráfico de funil (Figuras Suplementares S2-S4) não mostrou indícios de viés de publicação, com gráficos simétricos observados para os desfechos.

### Análise de sensibilidade

Realizamos uma análise de sensibilidade “leave-one-out” para avaliar a influência de estudos individuais nos resultados agrupados. A remoção de qualquer estudo individual teve impacto mínimo nos desfechos de eficácia. As análises de sensibilidade “leave-one-out” estão detalhadas nas Figuras Suplementares S25-S28.

### Análise de metarregressão

Para o tempo de fluoroscopia, a proporção de pacientes com FA persistente moderou significativamente os resultados (estimativa = -0,1295; IC 95% -0,1644 a -0,0945; p < 0,001), explicando toda a heterogeneidade observada (R^2^ = 100%) e indicando tempos de fluoroscopia mais curtos em estudos com maiores proporções de pacientes com FA persistente. Em contraste, para ausência de arritmias atriais, nenhum efeito moderador significativo foi observado (estimativa = -0,0006; IC 95% -0,0059 a 0,0048, p = 0,84), com heterogeneidade residual moderada remanescente (I^2^ = 46,96%). As análises de metarregressão são detalhadas na Figura Suplementar S8.

### Análise sequencial de testes

O tamanho da informação necessária (RIS) foi calculado com um risco de 5% de erro tipo I e um risco de 20% de erro tipo II. Para o tempo total de fluoroscopia (Figura S9 Suplementar), a curva Z cumulativa cruzou tanto o RIS de 279 participantes quanto os limites do monitoramento convencional, indicando evidência suficiente para este desfecho. O tempo do procedimento pele a pele excedeu 100% do RIS e cruzou os limites convencionais, indicando dados suficientes (Figura S10 Suplementar). Da mesma forma, para ausência de qualquer arritmia atrial, a curva Z cumulativa ultrapassou 100% do RIS, mas não cruzou os limites do monitoramento convencional, sugerindo que não há diferença significativa entre os grupos (Figura S11 Suplementar).

## Discussão

Nesta revisão sistemática e metanálise de quatro estudos observacionais retrospectivos incluindo 605 pacientes, avaliamos a eficácia e a segurança da PFA em comparação à ablação por RF com vHPSD em pacientes com FA. Nossos principais achados sugerem que tanto a PFA quanto a vHPSD foram igualmente eficazes na obtenção de isolamento das veias pulmonares, sem diferenças significativas entre as duas técnicas. O tempo do procedimento, no entanto, foi notavelmente maior com a vHPSD, enquanto a PFA foi associada a um tempo total de fluoroscopia mais longo. Não houve diferença quanto à ausência de arritmias atriais. Apesar dessas diferenças nas características do procedimento, ambas as intervenções apresentaram perfis de segurança comparáveis, sem diferenças significativas em complicações gerais, tamponamento cardíaco, reações no local de acesso vascular ou eventos de acidente vascular cerebral.

O ensaio ADVENT,^23^ o primeiro ensaio clínico randomizado comparando PFA com ablação por RF, fornece um contexto valioso para nossos achados, apesar de não abordar especificamente as técnicas de vHPSD. O ADVENT demonstrou que a PFA não foi inferior à ablação por RF convencional em termos de eficácia e segurança, corroborando a crescente base de evidências de que a PFA é uma alternativa viável aos métodos tradicionais de ablação térmica. Embora nossa metanálise tenha se concentrado na comparação da PFA com a abordagem mais recente de vHPSD, o estudo ADVENT reforça a aplicabilidade mais ampla da PFA no tratamento da FA.

Além disso, um estudo publicado recentemente por Santos et al. oferece uma perspectiva relevante sobre a comparação de técnicas emergentes de ablação para FA, avaliando a PFA versus ablação por RF vHPSD em um ambiente clínico do mundo real.^24^ Os autores destacam as vantagens distintas de cada modalidade, apoiando nossas descobertas de que ambas as técnicas demonstram eficácia comparável na obtenção do isolamento da veia pulmonar e exibem perfis de segurança semelhantes.

A ablação por radiofrequência PFA e vHPSD representam estratégias avançadas na ablação por cateter para FA.^24,25^ A PFA emprega eletroporação não térmica para atingir seletivamente o tecido cardíaco, minimizando danos colaterais às estruturas adjacentes, como o esôfago e as veias pulmonares^7^ Seu promissor perfil de segurança impulsionou a rápida adoção, particularmente na redução de complicações como estenose da veia pulmonar e lesão do nervo frênico. Em contraste, a ablação por RF de vHPSD utiliza energia de alta intensidade em um curto período, alcançando a formação precisa da lesão com maior eficiência e tempos de procedimento reduzidos.^4^ Esta metanálise se concentra na síntese de evidências para avaliar a eficácia do procedimento, a segurança e os resultados clínicos dessas técnicas, fornecendo insights para orientar a otimização de estratégias de ablação.

Os tempos de procedimento mais curtos associados à PFA provavelmente se devem ao seu design de disparo único e à sua fácil manobrabilidade, otimizando o fluxo de trabalho no laboratório de eletrofisiologia e aprimorando a experiência do paciente, conforme observado em estudos anteriores.^25,26^ Além dos tempos de procedimento mais curtos, o tempo de permanência no átrio esquerdo favoreceu significativamente a PFA. Este achado está alinhado com evidências emergentes na literatura que destacam a eficiência do procedimento com a PFA. Estudos têm demonstrado consistentemente que o design de disparo único e a abordagem de eletroporação direcionada da PFA otimizam o fluxo de trabalho e reduzem o tempo necessário para a manipulação do cateter dentro do átrio esquerdo, em comparação com as técnicas de ablação convencionais ou vHPSD, reduzindo o risco de complicações associadas à cateterização prolongada.^26,27^ Em contraste, as técnicas vHPSD dependem do uso eficiente de energia de alta potência para criar lesões grandes e uniformes, o que também apoia a eficiência do procedimento, mas não ultrapassa a velocidade proporcionada pelo PFA.^4-6^

No entanto, embora a PFA ofereça a vantagem de uma duração de procedimento mais curta, esse benefício é contrabalançado por sua associação com tempos de fluoroscopia mais longos, uma desvantagem significativa, dados os riscos da radiação ionizante tanto para pacientes quanto para profissionais de saúde com exposição crônica. Essa limitação pode ser decorrente da navegação por cateter mais complexa necessária durante a PFA, em comparação com as técnicas vHPSD, que frequentemente utilizam sistemas de mapeamento eletroanatômico para minimizar a dependência da fluoroscopia.^25,26^ A escolha entre ablação por PFA e vHPSD deve, portanto, ser individualizada, levando em consideração fatores específicos do paciente e a experiência do operador. Por exemplo, pacientes com maior risco de complicações relacionadas à sedação podem se beneficiar dos tempos de procedimento mais curtos associados à PFA, enquanto aqueles que necessitam de ablações lineares concomitantes podem se beneficiar mais dos tempos de fluoroscopia reduzidos oferecidos pela vHPSD.

Tecnologias emergentes podem enfrentar esse desafio. O estudo SPHERE Per-AF^28^ avaliou um novo sistema que combina mapeamento eletroanatômico de alta densidade com ablação de dupla energia (RF ou campo pulsado) usando um único cateter de ponta treliçada. O estudo demonstrou que essa abordagem combinada reduziu o tempo do procedimento e aumentou a eficiência da fluoroscopia. Esses resultados sugerem que a integração do mapeamento eletroanatômico em futuros sistemas de PFA pode reduzir ainda mais o tempo de fluoroscopia e melhorar a segurança do procedimento. Tais inovações podem consolidar a posição do PFA como o método preferencial para ablação de FA, especialmente em ambientes focados em fluxos de trabalho rápidos e eficientes.

Apesar dessas diferenças técnicas, a ausência de variação significativa na ausência de arritmias atriais entre os dois grupos sugere que ambas as abordagens são igualmente eficazes no controle da FA a curto e médio prazo. Essa descoberta está alinhada com pesquisas anteriores que indicam que, embora o PFA possa oferecer vantagens na consistência da lesão e na seletividade do tecido miocárdico, ele não supera consistentemente o vHPSD em termos de sobrevida livre de arritmias.^29,30^

Os resultados de segurança não apresentaram diferenças significativas entre os dois métodos. Especificamente, não houve variações substanciais nas taxas gerais de complicações, tamponamento cardíaco, reações no local de acesso vascular ou incidência de acidente vascular cerebral ou ataque isquêmico transitório (AIT). Esses achados sugerem que as técnicas de ablação por PFA e vHPSD apresentam perfis de segurança comparáveis, oferecendo segurança quanto à segurança geral de ambas as intervenções.^4,9^ No entanto, é importante notar que a ausência de diferenças significativas nesses resultados não descarta o potencial de eventos adversos raros ou não relatados.^31,32^ Considerando que ambos os procedimentos estão associados a baixas taxas de complicações, nossos resultados corroboram sua segurança. No entanto, mais ensaios clínicos randomizados e controlados em larga escala são necessários para compreender completamente o espectro de possíveis riscos e complicações, particularmente para eventos mais incomuns, porém graves, como lesão esofágica ou isquemia cerebral silenciosa.

Eventos adversos menores, como paralisia do nervo frênico, estenose da veia pulmonar, ulceração esofágica, vasoespasmo coronário e fístula átrio-esofágica, foram relatados esporadicamente nos estudos incluídos.^8-11^ No entanto, os dados disponíveis foram insuficientes para realizar uma análise robusta desses desfechos. Esses eventos, embora raros, podem ter implicações clínicas significativas e justificam consideração cuidadosa, principalmente porque nem sempre podem ser capturados em estudos retrospectivos menores. A escassez de relatos reforça a necessidade de estudos prospectivos maiores, com monitoramento padronizado de eventos adversos, para melhor avaliar a incidência e o impacto clínico dessas complicações. A compreensão desses eventos raros é fundamental para embasar a escolha da estratégia de ablação e garantir a segurança do paciente, principalmente porque as técnicas de PFA e vHPSD continuam a evoluir e a ganhar ampla adoção na prática clínica.

A análise de metarregressão indicou que a proporção de pacientes com FA persistente influenciou significativamente o tempo de fluoroscopia, com proporções maiores de FA persistente associadas a durações mais curtas da fluoroscopia. Esse achado sugere que características do paciente, como o tipo de FA, podem desempenhar um papel na eficiência do procedimento dessas intervenções, conforme observado na literatura.^33^ Uma explicação plausível é que os pacientes com FA persistente frequentemente têm necessidades mais extensas de modificação do substrato, levando os operadores a confiarem fortemente em sistemas avançados de mapeamento eletroanatômico em vez de fluoroscopia para orientar a ablação.^34-37^ Isso reduz a necessidade de exposição prolongada à fluoroscopia durante o procedimento.

Apesar dessas diferenças nos procedimentos, não foi observado efeito significativo na ausência de arritmias atriais, indicando que o tipo de FA não afeta substancialmente a eficácia a longo prazo dos procedimentos de ablação. Isso destaca o desempenho robusto das técnicas de ablação por PFA e vHPSD na obtenção de controle duradouro do ritmo, independentemente do tipo de FA. É importante ressaltar que a ausência de impacto nas taxas de recorrência ressalta a importância de adaptar as abordagens dos procedimentos para otimizar a segurança e a eficiência, mantendo a eficácia.

O TSA em nosso estudo indicou evidências suficientes para o tempo total de fluoroscopia e o tempo do procedimento pele a pele, visto que ambos os desfechos ultrapassaram os limites do RIS e do monitoramento convencional. Isso sugere achados confiáveis para esses desfechos, particularmente em relação à eficiência do PFA na redução dos tempos de procedimento. No entanto, para ausência de arritmias atriais, embora a curva Z cumulativa tenha ultrapassado 100% do RIS, ela não ultrapassou os limites convencionais, o que implica que as evidências para esse desfecho permanecem inconclusivas e requerem mais ensaios clínicos. Esses achados ressaltam a importância de pesquisas adicionais para confirmar os desfechos de arritmia em longo prazo e a robustez do PFA em comparação com a ablação por vHPSD.

Nosso estudo tem várias limitações. Primeiro, a inclusão de apenas estudos observacionais retrospectivos introduz o potencial de viés, particularmente na seleção de pacientes e no relato de desfechos. Embora tenhamos empregado critérios rigorosos para inclusão de estudos e avaliação de qualidade, a natureza observacional dos estudos incluídos limita a generalização de nossos resultados. Segundo, o período de acompanhamento nos estudos incluídos foi relativamente curto, limitando nossa capacidade de tirar conclusões sobre desfechos de longo prazo, como prevenção de AVC ou a necessidade de procedimentos repetidos. Por fim, os estudos variaram em termos das técnicas específicas usadas dentro do grupo vHPSD, o que pode ter influenciado os resultados. Embora tenhamos buscado minimizar essas variações por meio de nossos critérios de inclusão, pesquisas adicionais comparando formas específicas de vHPSD com PFA em um ambiente de ensaio clínico randomizado são necessárias para esclarecer esses achados.

## Conclusão

Esta metanálise, incluindo quatro estudos e 605 pacientes, demonstrou que a ablação por RF com PFA e vHPSD são opções eficazes e seguras para o tratamento da FA. A PFA foi associada a tempos de procedimento mais curtos, mas exigiu tempos de fluoroscopia mais longos. Não houve diferença na ausência de arritmias atriais e na incidência de complicações entre os grupos. Além disso, ensaios clínicos randomizados, bem conduzidos e em larga escala, são necessários para avaliar a segurança e a eficácia desses procedimentos a longo prazo.
