# Supplementary material for: Ablação por Campo Pulsado versus Ablação por Radiofrequência de Curta Duração e Potência Muito Alta na Fibrilação Atrial: Uma Revisão Sistemática e Metanálise
Source: Arq Bras Cardiol. 2025 Jul 29;122(7):e20240845. [Article in Portuguese] doi: 10.36660/abc.20240845 (PMC12695100; doi:10.36660/abc.20240845)
Supplement: Translation

# Translation: Pulsed Field Ablation Versus Very High-Power Short-Duration Radiofrequency Ablation in Atrial Fibrillation: A Systematic Review and Meta-Analysis

**DOI:** 10.36660/abc.20240845i

**Authors:** Asad Iqbal, Wellgner Fernandes Oliveira Amador, Martin Cevallos-Cueva, Maaz Ahmad, Juan Alejandro Pinilla Alarcón, Radamés Vieira Diniz

**Affiliations:** 1 Pakistan Bacha Khan Medical College, Mardan – Pakistan; 2 Brazil Universidade Federal de Campina Grande, Cajazeiras, PB – Brazil; 3 Ecuador School of Medicine, Universidad Central del Ecuador, Quito – Ecuador; 4 Colombia CES University, School of Medicine, Medellín – Colombia

**Keywords:** Catheter Ablation, Atrial Fibrillation, Efficacy, Safety

## Abstract

**Background:**

Pulmonary vein isolation (PVI) is a cornerstone treatment for atrial fibrillation (AF). Pulsed-field ablation (PFA) and very high-power short-duration (vHPSD) radiofrequency (RF) ablation are emerging technologies, but their comparative efficacy and safety remain unclear.

**Objectives:**

To assess the efficacy and safety of PFA compared to vHPSD RF ablation for AF.

**Methods:**

A systematic search of PubMed, Embase, Web of Science, and Cochrane databases identified studies comparing PFA and vHPSD ablation. Outcomes included PVI success, skin-to-skin procedure time, fluoroscopy time, freedom from atrial arrhythmias, and complications such as cardiac tamponade, stroke, and vascular access events. Continuous outcomes were analyzed using mean differences (MD), while binary outcomes were assessed with risk ratios (RR) and 95% confidence intervals (CI). A significance threshold of p < 0.05 was considered for statistical analyses. This study is registered in PROSPERO (CRD42024619301).

**Results:**

Four observational studies with 605 patients were included, of whom 315 (52%) underwent PFA. PFA and vHPSD achieved similar PVI success (RR 1.00; 95% CI 0.99–1.01; p = 1). PFA reduced procedure time (MD -30.07 min; 95% CI -31.41 to -28.74; p<0.01) but increased fluoroscopy time (MD 6.87 min; 95% CI 3.66–10.08; p<0.01). Freedom from atrial arrhythmias was comparable (RR 1.03; 95% CI 0.94–1.14; p=0.5). Complication rates, including cardiac tamponade, stroke, and vascular access issues, showed no significant differences between groups.

**Conclusion:**

PFA significantly shortens procedure time but requires longer fluoroscopy compared to vHPSD. Both techniques exhibit comparable efficacy for PVI and arrhythmia freedom, with similar safety profiles.

## Introduction

Atrial fibrillation (AF), the most common sustained cardiac arrhythmia, significantly impacts global healthcare due to its association with increased morbidity, mortality, and healthcare utilization.^1,2^ Catheter ablation has emerged as a cornerstone treatment for AF, offering rhythm control and symptomatic relief.^2,3^ Among contemporary ablation strategies, very high-power short-duration (vHPSD) radiofrequency (RF) ablation is valued for its efficiency and precision, while pulsed field ablation (PFA), a non-thermal technique utilizing electroporation, has gained attention for its potential to minimize collateral tissue damage.^4-6^

Since its introduction in Europe in 2021, PFA has been increasingly adopted for its promising safety profile, particularly in reducing the risk of complications such as pulmonary vein stenosis and damage to adjacent structures.^7^ However, despite these advancements, there remains a paucity of direct comparisons between PFA and vHPSD RF ablation, as existing studies are largely observational and retrospective in nature.^8-11^ This lack of randomized controlled trials limits the ability to establish definitive comparative efficacy and safety profiles.

To address this gap, this meta-analysis systematically synthesizes the available evidence to evaluate procedural and clinical outcomes associated with PFA and vHPSD RF ablation. By providing a comprehensive assessment of these techniques, including trial sequential analysis (TSA), this study seeks to inform clinical decision-making and support the optimization of ablation strategies for AF management in diverse patient populations. We chose vHPSD RF ablation as the comparator because it is the most recent evolution of thermal ablation, differing from HPSD by utilizing ultra-short, high-energy applications with active temperature control.

## Methods

This systematic review followed the recommendations of the Cochrane Collaboration^12^ and the Preferred Reporting Items for Systematic Reviews and Meta-Analysis (PRISMA) guidelines,^13^ including the design, implementation of the steps, analysis, and description of the results. The study protocol was registered in the International Prospective Register of Systematic Reviews (PROSPERO) under the number CRD42024619301.

### Search strategy

A systematic search on PubMed (MEDLINE), Embase, Cochrane Central, and Clinical Trial databases was conducted on November 25, 2024. The following medical subject heading terms have been included: ‘atrial fibrillation’, ‘af’, ‘afib’, ‘atrial fibrillation’, ‘a-fib’, ‘atrial flutter’, ‘cardiac arrhythmias’, ‘catheter ablation’, ‘ablation’, ‘pulsed field’, ‘pfa’, ‘hpsd’, ‘high power short duration’. The search strategy is detailed in Supplementary Table S1.

### Data extraction

After removing duplicates, two authors (A.I. and W.A.) screened the titles and abstracts, independently evaluating the full-text articles for inclusion based on pre-specified criteria. Discrepancies were discussed and resolved by a third reviewer (M.A.). Data extraction was conducted independently by A.I. and W.A., prioritizing information relevant to the study’s objective.

### Eligibility criteria

Eligible studies for this systematic review met the following criteria: (I) studies evaluating efficacy or safety without time restrictions; (II) inclusion of patients with atrial fibrillation; (III) interventions involving PFA; (IV) vHPSD ablation as control; and (V) reporting at least one outcome of interest. Exclusion criteria were: (I) overlapping populations, defined by shared institutions and recruitment periods; (II) populations outside the scope of interest; (III) republished literature; (IV) protocols without reported results; (V) reviews, case reports, case series, background articles, expert opinions, or in vivo/in vitro studies; (VI) duplicate data from the same clinical trial; or (VII) absence of a comparator group.

In the present study, the vHPSD RF ablation technique was defined based on parameters established in the literature.^4^ We considered vHPSD as procedures that used power ≥ 90W with durations ≤ 4 seconds per application, delivered through irrigated catheters with temperature control.

### Outcome measures and subgroup analysis

Efficacy outcomes were: (1) success in achieving pulmonary vein isolation, (2) skin-to-skin procedure time, (3) fluoroscopy time, (4) freedom from any atrial arrhythmia (atrial flutter, AF, and atrial tachycardia recurrences lasting at least 30 seconds during follow-up after a 1-month blanking period), and (5) left atrial dwell time. Safety outcomes included the (6) overall incidence of complications, (7) cardiac tamponade, (8) vascular access site reactions, and (9) stroke or TIA.

### Quality assessment

We assessed the risk of bias using the Cochrane ROBINS-I (Risk Of Bias In Non-randomised Studies—of Interventions) tool,^14^ which evaluates non-randomized studies of interventions across seven domains: confounding, selection of participants, classification of interventions, deviations from intended interventions, missing data, measurement of outcomes, and selection of reported results. The assessment was independently performed by two reviewers (A.I. and A.P.), with disagreements resolved by discussion or consultation with a third reviewer. Each domain was rated as having low, moderate, serious, or critical risk of bias, ensuring a comprehensive evaluation. The layout was produced by RobVis.^15^

### Certainty of evidence

Further, the Grading of Recommendations, Assessment, Development, and Evaluation (GRADE) tool was employed by two independent authors (W.A. and M.A.) using the GRADEpro Guideline Development Tool^16^ to evaluate the level of certainty of the evidence in this meta-analysis, with categorizations ranging from high to very low.^17^ Any disagreements were discussed and resolved through a consensus.

### Sensitivity analysis

The stability of the pooled estimates was assessed through a leave-one-out analysis, where data from each study were sequentially removed, and the remaining dataset re-analyzed. This helped ensure that any single study did not unduly influence the aggregated effect sizes.

### Statistical analysis

Statistical analysis was performed using R software and RStudio (version 2024.04.1+748; R Core Team, Vienna, Austria), employing DerSimonian and Laird’s random-effects model to calculate pooled analyses with 95% confidence intervals (CI).^18^ The results were presented as a pooled analysis in forest plots. Binary outcomes were assessed with risk ratios (RRs), continuous outcomes with mean differences (MDs), and results were displayed in forest plots. Heterogeneity was evaluated using the Cochrane Q chi-square test and I^2^ statistic, with P-values <0.10 and I^2^ >30% indicating significant heterogeneity.^19^ Publication bias was assessed with funnel plots.

### Meta-regression analysis

To evaluate the influence of the proportion of patients with persistent AF on procedural and clinical outcomes, meta-regression analyses were conducted. The percentage of patients with persistent AF was included as a covariate in the model to assess its potential impact on total fluoroscopy time and freedom from atrial arrhythmias, which were used as dependent variables. The analyses were performed using R software (version 4.4), with results reported as estimates and 95% confidence intervals. Statistical significance was determined at a threshold of p < 0.05.

### Trial sequential analysis

Trial sequential analysis (TSA) was performed using TSA software (version 0.9.5.10 beta)^20^ to assess sample size adequacy and determine the need for further research. Diversity-adjusted information size was calculated, accounting for variability between trials and sampling error, with a 5% type I error risk (α = 5%) and 20% type II error risk (β = 20%).^21,22^ Crossing the trial sequential monitoring boundary before reaching the required information size indicates conclusive evidence, whereas failure to cross it suggests the need for additional trials.

## Results

### Study selection

The initial search strategy yielded 1,094 results (Figure 1). After removing 607 duplicates, 487 articles were screened based on title and abstract according to the established inclusion and exclusion criteria. From this pool, 11 records were selected for full-text reading. Finally, this meta-analysis included four retrospective observational studies.^8-11^

**Figure 1:** – Preferred Reporting Items for Systematic Reviews and Meta-Analysis (PRISMA) flow diagram of study screening and selection.

### Baseline characteristics of included studies

The studies comprised a total of 605 patients, of whom 315 (52%) underwent PFA. The follow-up period extended up to 6 months. The mean age ranged from 61.6 to 67 years, with 250 (41%) being female. In addition, 234 (37%) of the participants had persistent atrial fibrillation. BMI ranged from 25.23 to 28 kg/m^2^. The most common comorbidities were hypertension (380, 62.8%), coronary artery disease (93, 17.3%), and diabetes mellitus (33, 12.5%). Baseline characteristics of the included studies are detailed in Table 1.

**Table 1:** – Baseline characteristics of included studies

Studies	Follow-up (months)	Sample size		Female, n (%)		Age, years (SD)		Persistent AF, n (%)		Paroxysmal AF, n (%)		BMI, kg/m^2^ (SD)		LVEF, % (SD)		DM, n (%)		CHA2DS2-VASc SCORE (SD)		Congestive HF, n (%)		CAD, n (%)		HTN, n (%)	
		PFA	vHPSD	PFA	vHPSD	PFA	vHPSD	PFA	vHPSD	PFA	vHPSD	PFA	vHPSD	PFA	vHPSD	PFA	vHPSD	PFA	vHPSD	PFA	vHPSD	PFA	vHPSD	PFA	vHPSD
Dello Russo 2024	6	171	171	64 (37)	60 (35)	64.3 (7.5)	65.3 (9)	56 (33)	54 (32)	115 (67)	117 (68)	25.23 (1.12)	25.23 (1.12)	57.4 (5.2)	58.2 (3.7)	NA	NA	2 (1.5)	2 (1.5)	46 (27)	41 (24)	24 (14)	22 (13)	105 (61)	106 (62)
South 2024	6	52	30	15 (29)	16 (53)	67 (10)	73 (7)	27 (52)	17 (57)	25 (48)	13 (43)	28 (6)	27 (6)	52 (8)	47 (14)	8 (15)	4 (13)	4 (1.6)	4 (1.7)	20 (38)	13 (43)	9 (17)	13 (43)	43 (83)	25 (83)
Wormann 2023	6	57	57	38 (67)	34 (60)	67 (13)	67 (12)	40 (70)	40 (70)	17 (30)	17 (30)	28 (5)	27 (4)	56 (6)	56 (9)	9 (16)	8 (14)	3 (NA)	3 (NA)	NA	NA	14 (25)	11 (19)	37 (65)	34 (60)
Popa 2023	6	35	32	11 (31)	12 (37.5)	61.6 (11.4)	62.2 (12.8)	0 (0)	0 (0)	35 (100)	32 (100)	26.7 (4.9)	25.4 (2.7)	58.5 (2.7)	57.9 (7.8)	1 (2.9)	3 (9.4)	1.7 (1.7)	2.0 (1.4)	NA	NA	NA	NA	14 (40)	16 (50)

Continuous variables are presented as mean at baseline (Standard Deviation, SD). NA: not available; SD: standard deviation; AF: atrial fibrillation; BMI: body mass index; LVEF: left ventricular ejection fraction; DM: diabetes mellitus; HF: heart failure; CAD: coronary artery disease; HTN: hypertension. All included studies adopted a statistical significance level of α = 0.05. Source: Authors.

### Pooled analysis of all studies

A pooled analysis of four studies demonstrated that both interventions achieved pulmonary vein isolation with equal effectiveness (RR 1.00; 95% CI 0.99–1.01 [Figure 2A]). However, the skin-to-skin procedure time was longer in the vHPSD ablation group (MD -31.41 min; 95% CI -31.41 to -28.74 [Figure 2B]), while total fluoroscopy time was greater in the PFA group (MD 6.87 min; 95% CI 3.66–10.08 [Figure 2C]). No significant difference was observed in freedom from any atrial arrhythmias (RR 1.03; 95% CI 0.94–1.14 [Figure 2D]). Notably, left atrial dwell time favored the PFA intervention (MD -22.15 min; 95% CI -31.15 to -14.3 [Figure 2E]).

**Figure 2:** – Forest plots for (A) pulmonary vein isolation; (B) skin-to-skin procedure time; (C) total fluoroscopy time; (D) freedom from atrial arrhythmias; and (E) left atrial dwell time. Source: Authors.

The safety profiles of the two interventions were comparable. There were no significant differences in the incidence of overall complications (RR 1.05; 95% CI 0.51–2.16 [Figure 3A]), cardiac tamponade (RR 5.00; 95% CI 0.25–101.87 [Figure 3B]), vascular access site reactions (RR 1.29; 95% CI 0.41–4.06 [Figure 3C]), or stroke (RR 0.73; 95% CI 0.05–9.83 [Figure 3D]).

**Figure 3:** – Forest plots for (A) any complications; (B) cardiac tamponade; (C) vascular access site complications; and (D) stroke. Source: Authors.

### Risk of bias within studies

As detailed in Supplementary Fig. S1, three studies^8^ were assessed as having a moderate risk of bias. This was predominantly attributed to issues in confounding, participant selection, intervention classification, and outcome measurement. One study^11^ was categorized as having a high risk of bias, primarily due to confounding.

### Certainty of evidence and publication bias

According to the GRADE criteria (Supplementary Table S2), the certainty of evidence was very low for all outcomes assessed. This was primarily attributed to the non-randomized design of the included studies and their moderate to high risk of bias. Funnel plot analysis (Supplementary Figs. S2-S4) showed no indications of publication bias, with symmetrical plots observed for outcomes.

### Sensitivity analysis

We performed leave-one-out sensitivity analysis to assess the influence of individual studies on the pooled results. The removal of any single study had minimal impact on the outcomes for efficacy endpoints. Leave-one-out sensitivity analyses are detailed in Supplementary Figures S25-S28.

### Meta-regression analysis

For fluoroscopy time, the proportion of persistent AF patients significantly moderated the results (estimate = -0.1295; 95% CI -0.1644 to -0.0945; p < 0.001), explaining all observed heterogeneity (R^2^ = 100%) and indicating shorter fluoroscopy times in studies with higher proportions of persistent AF patients. In contrast, for freedom from atrial arrhythmias, no significant moderating effect was observed (estimate = -0.0006; 95% CI -0.0059 to 0.0048, p = 0.84), with moderate residual heterogeneity remaining (I^2^ = 46.96%). Meta-regression analyses are detailed in Supplementary Figure S8.

### Trial sequential analysis

The required information size (RIS) was calculated with a 5% risk of type I error and a 20% risk of type II error. For total fluoroscopy time (Supplementary Figure S9), the cumulative Z-curve crossed both the RIS of 279 participants and the conventional monitoring boundaries, indicating sufficient evidence for this endpoint. Skin-to-skin procedure time exceeded 100% of the RIS and crossed conventional boundaries, indicating sufficient data (Supplementary Figure S10). Similarly, for freedom from any atrial arrhythmia, the cumulative Z-curve surpassed 100% of the RIS but did not cross the conventional monitoring boundaries, suggesting no significant difference between groups (Supplementary Figure S11).

## Discussion

In this systematic review and meta-analysis of four retrospective observational studies including 605 patients, we evaluated the efficacy and safety of PFA compared to vHPSD RF ablation in patients with AF. Our main findings suggest that both PFA and vHPSD were similarly effective in achieving PVI, with no significant differences between the two techniques. The procedure time, however, was notably higher with vHPSD, while PFA was associated with a longer total fluoroscopy time. There was no difference regarding the freedom from atrial arrhythmias. Despite these differences in procedural characteristics, both interventions had comparable safety profiles, with no significant differences in overall complications, cardiac tamponade, vascular access site reactions, or stroke events.

The ADVENT trial,^23^ the first randomized clinical trial comparing PFA with radiofrequency ablation, provides valuable context for our findings, despite not specifically addressing vHPSD techniques. ADVENT demonstrated that PFA was non-inferior to conventional RF ablation in terms of efficacy and safety, supporting the growing evidence base for PFA as a viable alternative to traditional thermal ablation methods. While our meta-analysis focused on comparing PFA with the more recent vHPSD approach, the ADVENT trial reinforces the broader applicability of PFA in AF management.

Additionally, a recently published study by Santos et al. offers a relevant perspective on the comparison of emerging ablation techniques for atrial fibrillation, evaluating pulsed field ablation versus HPSD RF ablation in a real-world clinical setting.^24^ The authors highlight the distinct advantages of each modality, supporting our findings that both techniques demonstrate comparable efficacy in achieving pulmonary vein isolation and exhibit similar safety profiles.

PFA and vHPSD radiofrequency ablation represent advanced strategies in catheter ablation for AF.^24,25^ PFA employs non-thermal electroporation to selectively target cardiac tissue, minimizing collateral damage to adjacent structures such as the esophagus and pulmonary veins.^7^ Its promising safety profile has driven rapid adoption, particularly for reducing complications like pulmonary vein stenosis and phrenic nerve injury. In contrast, vHPSD RF ablation uses high-intensity energy over a short duration, achieving precise lesion formation with enhanced efficiency and reduced procedure times.^4^ This meta-analysis focuses on synthesizing evidence to evaluate the procedural efficacy, safety, and clinical outcomes of these techniques, providing insights to guide the optimization of ablation strategies.

The shorter procedure times associated with PFA are likely due to its single-shot design and easy maneuverability, optimizing workflow in the electrophysiology lab and enhancing the patient experience, as observed in previous studies.^25,26^ In addition to shorter procedure times, left atrial dwell time significantly favored PFA. This finding aligns with emerging evidence in the literature highlighting the procedural efficiency of PFA. Studies have consistently demonstrated that the single-shot design and targeted electroporation approach of PFA streamline workflow and reduce the time required for catheter manipulation within the left atrium, as compared to conventional or vHPSD ablation techniques, lowering the risk of complications associated with prolonged catheterization.^26,27^ In contrast, vHPSD techniques rely on the efficient use of high-power energy to create large, uniform lesions, which also supports procedural efficiency but does not surpass the speed afforded by PFA.^4-6^

However, while PFA offers the advantage of shorter procedural duration, this benefit is counterbalanced by its association with longer fluoroscopy times, a significant drawback given the risks of ionizing radiation for both patients and healthcare professionals with chronic exposure. This limitation may stem from the more complex catheter navigation required during PFA, compared to vHPSD techniques, which frequently utilize electroanatomic mapping systems to minimize fluoroscopy dependency.^25,26^ The choice between PFA and vHPSD ablation should therefore be individualized, taking into account patient-specific factors and operator expertise. For example, patients at higher risk for sedation-related complications may favor the shorter procedure times associated with PFA, whereas those needing concomitant linear ablations might benefit more from the reduced fluoroscopy times offered by vHPSD.

Emerging technologies may address this challenge. The SPHERE Per-AF study^28^ evaluated a novel system combining high-density electroanatomic mapping with dual-energy ablation (RF or pulsed field) using a single lattice-tip catheter. The study showed that this combined approach reduced procedure times and enhanced fluoroscopy efficiency. These results suggest that integrating electroanatomic mapping into future PFA systems could lower fluoroscopy time further and improve procedural safety. Such innovations may solidify PFA’s position as the preferred method for AF ablation, especially in environments focused on efficient, rapid workflows.

Despite these technical differences, the lack of significant variation in freedom from atrial arrhythmias between the two groups suggests that both approaches are similarly effective in controlling AF over the short to medium term. This finding aligns with previous research indicating that while PFA may offer advantages in lesion consistency and myocardial tissue selectivity, it does not consistently outperform vHPSD in terms of arrhythmia-free survival.^29,30^

The safety outcomes showed no significant differences between the two methods. Specifically, there were no substantial variations in overall complication rates, cardiac tamponade, vascular access site reactions, or the incidence of stroke or transient ischemic attack (TIA). These findings suggest that both PFA and vHPSD ablation techniques exhibit comparable safety profiles, offering reassurance regarding the overall safety of both interventions.^4,9^ However, it is important to note that the absence of significant differences in these outcomes does not rule out the potential for rare or unreported adverse events.^31,32^ Given that both procedures are associated with low complication rates, our results support their safety. However, further large-scale, randomized controlled trials are necessary to fully understand the spectrum of possible risks and complications, particularly for more uncommon but serious events such as esophageal injury or silent cerebral ischemia.

Minor adverse events, such as phrenic nerve palsy, pulmonary vein stenosis, esophageal ulceration, coronary vasospasm, and atrial-esophageal fistula, were reported sporadically in the included studies.^8-11^ However, the data available were insufficient to perform a robust analysis of these outcomes. These events, though rare, can have significant clinical implications and warrant careful consideration, particularly as they may not always be captured in smaller, retrospective studies. The limited reporting underscores the need for larger, prospective trials with standardized adverse event monitoring better to evaluate the incidence and clinical impact of these complications. Understanding these rare events is critical to informing the choice of ablation strategy and ensuring patient safety, particularly as both PFA and vHPSD techniques continue to evolve and gain wider adoption in clinical practice.

Meta-regression analysis indicated that the proportion of patients with persistent AF significantly influenced fluoroscopy time, with higher proportions of persistent AF associated with shorter fluoroscopy durations. This finding suggests that patient characteristics, such as the type of AF, may play a role in the procedural efficiency of these interventions, as observed in the literature.^33^ One plausible explanation is that patients with persistent AF often have more extensive substrate modification requirements, leading operators to rely heavily on advanced electroanatomic mapping systems rather than fluoroscopy to guide ablation.^34-3 7^ This reduces the need for prolonged fluoroscopy exposure during the procedure.

Despite these procedural differences, no significant effect was observed on freedom from atrial arrhythmias, indicating that the type of AF does not substantially affect the long-term efficacy of the ablation procedures. This highlights the robust performance of both PFA and vHPSD ablation techniques in achieving durable rhythm control, regardless of AF type. Importantly, the lack of impact on recurrence rates underscores the importance of tailoring procedural approaches to optimize safety and efficiency while maintaining efficacy.

TSA in our study indicated sufficient evidence for total fluoroscopy time and skin-to-skin procedure time, as both outcomes crossed the RIS and conventional monitoring boundaries. This suggests reliable findings for these endpoints, particularly regarding the efficiency of PFA in reducing procedure times. However, for freedom from atrial arrhythmias, while the cumulative Z-curve surpassed 100% of the RIS, it did not cross conventional boundaries, implying that the evidence for this outcome remains inconclusive and requires further trials. These findings underscore the importance of additional research to confirm long-term arrhythmia outcomes and the robustness of PFA compared to vHPSD ablation.

Our study has several limitations. First, the inclusion of only retrospective observational studies introduces the potential for bias, particularly in patient selection and reporting of outcomes. Although we employed rigorous criteria for study inclusion and quality assessment, the observational nature of the included studies limits the generalizability of our results. Second, the follow-up period in the included studies was relatively short, limiting our ability to draw conclusions about long-term outcomes such as stroke prevention or the need for repeat procedures. Lastly, the studies varied in terms of the specific techniques used within the vHPSD group, which may have influenced the results. While we aimed to minimize these variations through our inclusion criteria, further research comparing specific forms of vHPSD with PFA in a randomized controlled trial setting is needed to clarify these findings.

## Conclusion

This meta-analysis, including four studies and 605 patients, showed PFA and vHPSD RF ablation to be both effective and safe options for treating AF. PFA was associated with shorter procedure times but required longer fluoroscopy times. There was no difference in freedom from atrial arrhythmias and incidence of complications between the groups. Further, larger-scale, well-conducted randomized controlled trials are needed to assess the long-term safety and efficacy of these procedures.
